# The Emergence of Cryptococcemia in COVID-19 Infection: A Case Report

**DOI:** 10.7759/cureus.19761

**Published:** 2021-11-20

**Authors:** Yerandy Gil, Yusleidi D Gil, Theodore Markou

**Affiliations:** 1 Internal Medicine, Saint Clare’s Health, Denville, USA; 2 Infectious Disease, Saint Clare’s Health, Denville, USA

**Keywords:** opportunistic infection, immunocompetent, covid-19, dexamethasone, cryptococcus neoformans (c. neoformans)

## Abstract

Cryptococcus neoformans is a fungus that can cause pulmonary, central nervous system, and dermatological infections, especially in an immunocompromised patient. This is a case report of a patient, who was presumptively immunocompetent that developed isolated cryptococcemia while being treated for coronavirus disease 19 (COVID-19) infection.

We report a case of a 59-year-old Hispanic man with a past medical history of hypertension, well-controlled diabetes mellitus, and class I obesity who was admitted for severe acute respiratory distress syndrome coronavirus 2 (SARS-COV-2) and subsequently was diagnosed with cryptococcal fungemia. The patient received 21 days of dexamethasone and during this period, blood and fungal cultures grew C. neoformans. The patient was alert and oriented, did not have focal neurological deficits or meningeal irritation signs; nonetheless, a lumbar puncture was attempted, but not successful. He was treated with intravenous amphotericin B for two weeks, followed by oral fluconazole for six weeks. Repeat blood cultures demonstrated clearance and he improved clinically.

In conclusion, this case report describes the possibility of an association between the use of dexamethasone in COVID-19 patients and the development of cryptococcal fungemia. In the review of literature, rare case reports worldwide have discussed this topic. This is clinically challenging as the emergence of opportunistic infections in these debilitated hosts can be detrimental. Maintaining a high clinical suspicion for this opportunistic infection while treating COVID-19 patients is necessary to prevent further mortality associated with this pandemic.

## Introduction

Albeit rare in developed countries, Cryptococcus neoformans or gattii cause approximately 220,000 infections per year worldwide. Infection is rarely seen in immunocompetent individuals, it is much more common in the immunocompromised population [1]. Transmission occurs through inhalation of spores, which can later lead to infections in the pulmonary, dermatological, or central nervous systems particularly in patients with impaired cell-mediated immunity [2]. Severe acute respiratory syndrome coronavirus 2 (SARS-COV-2) caused by coronavirus disease 19 (COVID-19) usually manifests as a respiratory infection that triggers an exaggerated immune response in its hosts [3]. We present a rare case of cryptococcal fungemia in the setting of dexamethasone use for hypoxemic respiratory failure in a patient with SARS-CoV-2 infection.

## Case presentation

**Figure 1:**
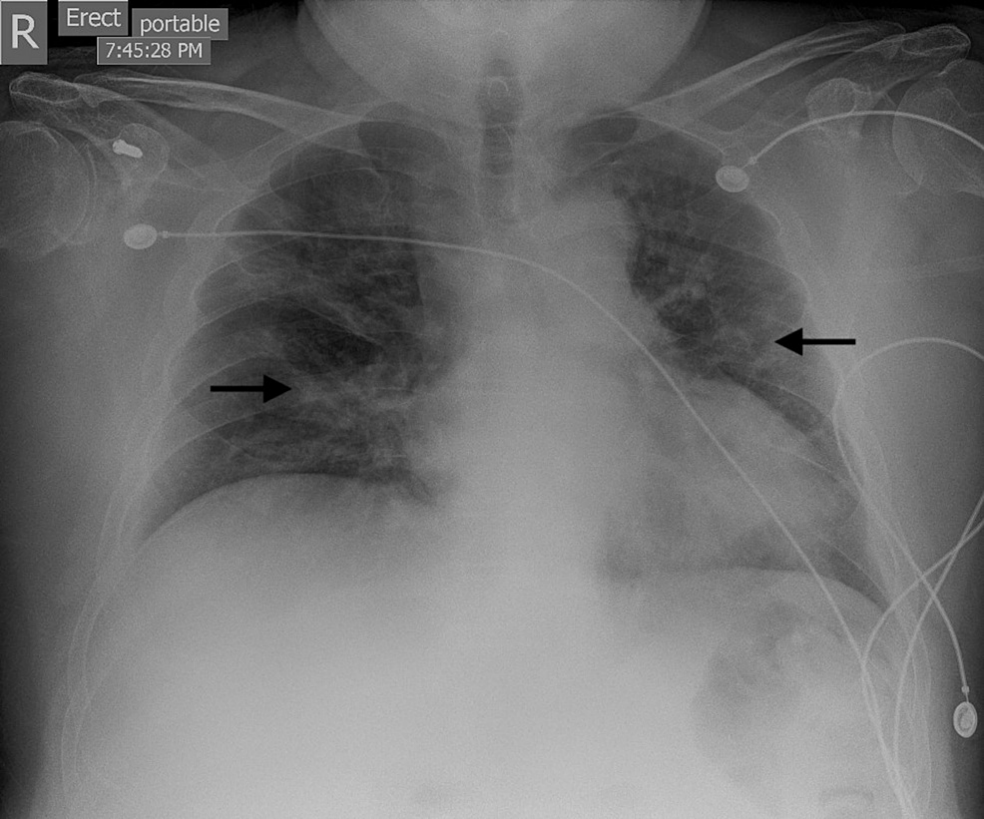
Admission chest x-ray demonstrating bilateral pulmonary infiltrates (arrows).

The patient is a 59-year-old male who had recently immigrated to the United States from Costa Rica with a past medical history of chronic hypertension, well-controlled diabetes mellitus (hemoglobin A1C 6.9%), and class I obesity who presented to the emergency department due to fever, shortness of breath, non-bilious, non-bloody emesis and abdominal/back pain of 24 hours evolution. Vital signs were significant for a temperature of 39.1 degrees Celsius and a respiratory rate of 29 breaths per minute. Physical exam revealed an obese gentleman in mild respiratory distress that was alert and oriented to person, place, and time, without focal neurological deficits and negative Kernig and Brudzinski signs; lungs were clear to auscultation bilaterally and his abdomen was protuberant, soft, non-distended, with normal bowel sounds and epigastric tenderness. The remainder of the examination was normal. His chest radiograph was noted to have bilateral extensive pulmonary infiltrates, but did not show any pulmonary nodules, cavitary lesion, or mass (Figure 1).

**Figure 2:**
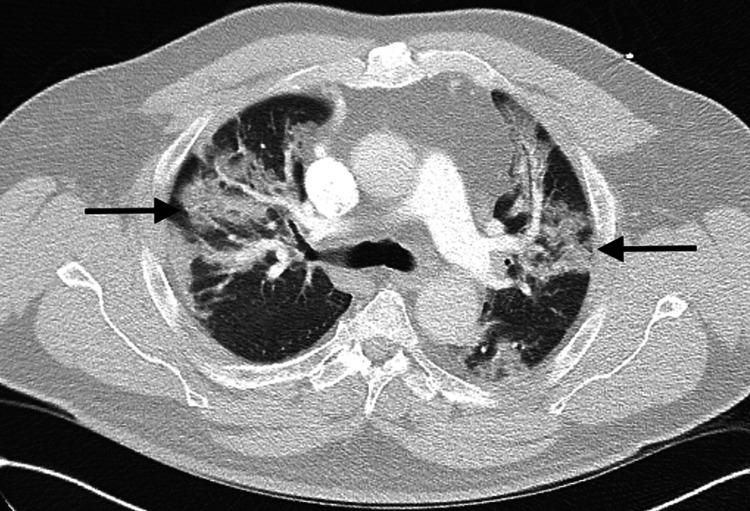
Chest CT demonstrating extensive bilateral ground glass infiltrates (arrows).

He was unvaccinated for COVID-19 infection and tested positive for SARS-CoV-2 via fluorescent immunoassay antigen test; a confirmatory test was not performed due to high clinical and radiographic suspicion in the setting of worldwide supply shortage. He was noted to be hypoxic on admission, requiring oxygen supplementation via nasal cannula, subsequently, his oxygen requirements rapidly increased over the first five days of hospitalization. The admission set of blood cultures were negative. He was treated with a seven-day course of intravenous (IV) azithromycin and ceftriaxone, remdesivir 200 mg IV initially, followed by 100 mg IV daily for four days [4], 6 mg of dexamethasone daily for 10 days, and then slowly tapered over the course of 21 days [5]. He did not receive monoclonal antibodies or other immune-modulating therapy. One week into his admission he had a respiratory failure that led to cardiopulmonary arrest with successful resuscitation and intubation. On hospital day 10, he developed fevers and was started on empiric antibiotic therapy with IV meropenem while blood, urine, and sputum cultures were being processed. C. neoformans was isolated from the blood cultures, which was confirmed by fungus culture and cryptococcal matrix-assisted laser desorption ionization-time-of-flight (MALDI-TOF) mass spectrometry. Interestingly, a latex agglutination test for cryptococcal antigen was negative. A lumbar puncture was attempted by interventional radiology but was unsuccessful after multiple attempts. Despite the absence of meningeal signs, further imaging with computed tomography (CT) of the head without contrast was pursued to assess for neurological involvement. It revealed no infarct, hemorrhage, masses, or hydrocephalus. On further examination, there were no dermatological lesions noted and CT of the chest was only consistent with bilateral ground glass pulmonary infiltrates (Figure 2).

A transthoracic echocardiogram demonstrated normal systolic and diastolic function with an ejection fraction (EF) > 55%, mild mitral regurgitation, no wall motion abnormalities, and no vegetations. A human immunodeficiency virus antigen/antibody test was non-reactive with a CD4+ T-cell count of 636 cells/microliter. Immunoglobulin concentrations were not measured. The corticosteroids were discontinued at the time of cryptococcal diagnosis and he was treated with IV amphotericin B liposome 5 mg/kg daily for two weeks, followed by oral fluconazole 800 mg daily for six weeks; with repeat blood cultures after therapy demonstrating fungal clearance with subsequent clinical improvement. Despite this management, he experienced a prolonged hospital stay, but eventually was weaned off mechanical ventilation and was discharged.

## Discussion

Cryptococcus may present as a pulmonary, skin, or central nervous system infection. Diagnosis is made through obtaining a detailed history, thorough physical exam, and appropriate imaging/laboratory testing. Fungal blood cultures, serum cryptococcal antigen, lumbar puncture, and biopsy of suspicious lesions are all helpful in confirming the diagnosis. Systemic antifungal therapy is the cornerstone of treatment: Azole therapy may be used for mild to moderate disease, however, induction with amphotericin B liposome and flucytosine is typically used for severe and/or life-threatening illness followed by consolidation with azole therapy [2].

The RECOVERY trial showed a decrease in the mortality of COVID-19 with the use of dexamethasone by suppressing the pro-inflammatory effects of SARS-CoV-2 [5]. Cryptococcal infection is rarely seen in the immunocompetent population; however, with the growing use of corticosteroids in the management of SARS-CoV-2 infection, this is a disease that clinicians need to be mindful of. Glucocorticoids dampen the immune system by diminishing the innate response via decreasing lymphocyte proliferation and T-cell activation, which inhibits cytokine release resulting in an impaired cell-mediated response. A prolonged course of corticosteroid therapy may suppress the immune system and thus lead to opportunistic infections such as cryptococcus. The concomitant use of corticosteroids and systemic antifungal medication is strongly discouraged and should be evaluated when considering the management of this complication, due to concerns for increased adverse events and delayed fungal clearance [6]. Conversely, the use of monoclonal antibodies, like those used in the Regen-Cov trial, can reduce hospitalization rates in COVID-19, leading to avoidance of steroids and thereby preventing an increased risk of opportunistic infections, such as cryptococcus [7].

As reported by Khatib et al., their case also developed cryptococcemia while undergoing treatment with corticosteroids for COVID-19. In contrast, they chose to use methylprednisone and hydrocortisone in conjunction with tocilizumab. Interestingly, both of their case and ours have similar patient comorbidities and neither patient had an overt reason for immunosuppression aside from the use of corticosteroids and/or immune modulator therapy. They were also unable to perform a lumbar puncture due to the patient's instability and their outcome was different despite using the standard treatment for cryptococcemia [8]. Thyagarajan, Mondy, and Rose, also presented a very similar case in regards to both demographics and hospital course that demonstrates an incidental finding of cryptococcal fungemia in a patient with COVID-19 pneumonia that despite aggressive means of oxygen supplementation did not improve clinically and was transitioned to comfort care. They also propose that reactivation of latent cryptococcal infection in an immunocompromised host is a less-likely means of acquiring the disease [9].

Alegre-Gonzalez et al. reported a case of a patient with disseminated cryptococcosis diagnosed on day 75 of COVID-19 infection in the setting of a prolonged course of both IV and oral steroids [10]. A case of cryptococcal meningoencephalitis with absent neurological symptoms is presented by Ghanem and Sivasubramanian [11]. These two cases highlight the importance of expanding the differential diagnosis to also include superimposed fungal infections in cases of COVID-19 with prolonged hospital courses.

## Conclusions

Our case demonstrates a new and emerging complication of an opportunistic infection associated with the treatment of SARS-CoV-2 infection. A review of the literature shows an increasing number of cases of cryptococcosis in patients who received corticosteroids and immune-modulating therapy in the setting of COVID-19 infection. This case, in conjunction with the literature review, draws attention to the possibility that low-dose corticosteroid therapy used in the management of COVID-19 may be sufficient to cause fungal infections in even immunocompetent hosts. It is important to recognize this potential complication as cryptococcosis carries a high mortality risk, particularly in patients with prolonged hospital courses due to COVID-19 pneumonia. Given limited studies on this topic, further investigation is needed to better understand the role of passive or active opportunistic infection surveillance while treating SARS-CoV-2 infection.
